# A general approach to simultaneous model fitting and variable elimination in response models for biological data with many more variables than observations

**DOI:** 10.1186/1471-2105-9-195

**Published:** 2008-04-15

**Authors:** Harri T Kiiveri

**Affiliations:** 1 CSIRO Mathematical and Information Sciences, The Leeuwin Center, 65 Brockway Road, Floreat, 6014, Western Australia

## Abstract

**Background:**

With the advent of high throughput biotechnology data acquisition platforms such as micro arrays, SNP chips and mass spectrometers, data sets with many more variables than observations are now routinely being collected. Finding relationships between response variables of interest and variables in such data sets is an important problem akin to finding needles in a haystack. Whilst methods for a number of response types have been developed a general approach has been lacking.

**Results:**

The major contribution of this paper is to present a unified methodology which allows many common (statistical) response models to be fitted to such data sets. The class of models includes virtually any model with a linear predictor in it, for example (but not limited to), multiclass logistic regression (classification), generalised linear models (regression) and survival models. A fast algorithm for finding sparse well fitting models is presented. The ideas are illustrated on real data sets with numbers of variables ranging from thousands to millions. R code implementing the ideas is available for download.

**Conclusion:**

The method described in this paper enables existing work on response models when there are less variables than observations to be leveraged to the situation when there are many more variables than observations. It is a powerful approach to finding parsimonious models for such datasets. The method is capable of handling problems with millions of variables and a large variety of response types within the one framework. The method compares favourably to existing methods such as support vector machines and random forests, but has the advantage of not requiring separate variable selection steps. It is also works for data types which these methods were not designed to handle. The method usually produces very sparse models which make biological interpretation simpler and more focused.

## Background

Many statistical models for studying the relationship between a response variable and a set of predictor variables have been developed over the years, e.g. generalised linear models [1], survival models [2] and multi class logistic regression models [3]. These models typically assume that there are many more *observations *than variables. However, with the advent of high throughput biotechnology data such as that collected by microarrays, SNP chips and mass spectrometers, it has become possible to gather data sets with several orders of magnitude more *variables *than observations. In this paper we describe a unified mechanism for enabling the use of a wide variety of existing statistical models in the case that there are many more variables than observations. Underlying this mechanism is a notion of model sparsity and the mechanism can be viewed as either likelihood based methodology with a sparsity penalty or a Bayesian methodology with a sparsity prior. There is some expositional advantage to the Bayesian approach so we will focus on that here. Fully Bayesian approaches to this problem do not seem tractable for the problem sizes to be considered.

The general approach and algorithm is described in the Results section below along with comments on practical implementation, and a number of real life examples of application of the method. The numbers of variables involved in these examples range from thousands to millions. Additional insight as to how the algorithm functions is described in Additional file 1 for the case of linear regression.

Before embarking on the description of the approach, we first introduce a small amount of notation. In the following we have N samples, and vectors such as y, z and μ have components y_i_, z_i _and μ_i _for i = 1,..., N. Vector multiplication and division is defined component wise and Δ (·) denotes a diagonal matrix whose diagonals are equal to the argument. We also use ||·|| to denote the Euclidean norm, and the L1 norm of a vector x is $\sum_{i}\left|x_{i}\right|$.

### Context and methods

We suppose that we are given an N by p matrix of data *X *with the number of variables p possibly, but not necessarily, much greater than the number of samples N. Associated with this matrix is a response vector *y*, and we are interested in building a predictive relationship between *y *and *X*. Such data matrices commonly occur with data collected from microarrays, SNP chips, and mass spectrometers. Let *L*(*y*|*X*, *β*, *φ*) be a likelihood function for a model we would like to fit to this data in order to achieve this. Here *β *is a p by 1 vector of parameters of primary interest, and *φ *a q by 1 vector of parameters of secondary interest, such as scale parameters. Example models include generalised linear models, multi-class logistic regression and proportional hazards survival models, however the discussion to follow is not limited to these models. We will describe a general method for simultaneous parameter estimation and variable selection which will cope with variable numbers in the order of millions for a wide variety of common (statistical) response models.

We begin with a Bayesian perspective, and specify a prior for the p by 1 parameter vector *β*, which attempts to capture the notion that most of the components of *β *are likely to be zero or at least "negligible". We then maximise the posterior distribution of the parameters of interest to get estimates of *β*. To define the prior consider a two step process. First we generate a variance from a distribution with the property that there is a high probability that the variance will be "very small". Given this variance, we then generate a parameter value from a normal distribution with this variance and mean value zero. Applying this process independently for each component of *β*, the marginal distribution of *β*, which we use as our prior, can be written

(1) $$p(\beta )=\int_{\nu^{2}}p\left(\beta |\nu^{2}\right)p\left(\nu^{2}\right)d\nu^{2}$$

where *p*(*β *|*ν*^2^) is *N*(0, Δ(*ν*^2^)). For this article we chose $p(\nu^{2})=\prod_{i=1}^{p}p(\nu_{i}^{2})$ where each of the $\nu_{i}^{2}$ has a scaled gamma distribution with common shape parameter 0 ≤ *k *≤ 1, and scale parameter b > 0. We will refer to this prior as the normal-gamma or NG prior below. This choice has worked very well in practice, however the methods do not depend on this choice. Some other possible choices are discussed in the supplementary information, see also Griffin and Brown [4]. We choose an uninformative prior for φ.

The prior for *β *can be written as a product of components of the form

(2) $$\begin{array}{cc} p(\beta_{i})=(\frac{2^{\left(0.5-k\right)}}{\sqrt{\pi }\Gamma (k)})\frac{\delta K_{0.5-k}(\delta |\beta_{i}|)}{{\left(\delta |\beta_{i}|\right)}^{\left(0.5-k\right)}}, & \delta =\sqrt{\frac{2}{b}} \end{array}$$

where *K *denotes a modified Bessel function of the third kind [5], and Γ denotes the gamma function. Some interesting special cases of this prior are:

(i) k = 1

(3) *p*(*β *_*i*_) = (*δ */2) exp(*δ *|*β *_*i*_|).

This prior is used in the Lasso [6], and enables an L1 constraint to be imposed on the parameters in the model. However, for k < 1 the prior is stronger than the Lasso "prior" and we focus attention on these priors here. Note that reliable, very sparse models are of particular interest in the development of diagnostics for disease.

(ii) k = 0

(4) *p*(*β *_*i*_) ∝ *δ *exp {-*δ *|*β *_*i*_|}/*δ *|*β *_*i*_|

(iii) k = 0, *δ *= 0,

(5) *p*(*β *_*i*_) ∝ 1/|*β *_*i*_|

This prior corresponds to using a Jeffreys hyperprior for the variances *ν*^2^, see Figueiredo [7,8] and Kiiveri [9].

In our Bayesian framework the posterior distribution of *β*, φ and *ν *given *y *is

(6) *p*(*β*, *φ*, *ν*|*y*) ∝ *L*(*y*|*β*, *φ*) *p*(*β *|*v*)*p*(*v*).

By treating *ν *as a vector of missing data we can use the EM algorithm [10] to maximise the log of *p*(*β*, *φ *|*y*) to produce maximum a posteriori (MAP) estimates of *β *and *φ*. This approach gets around the issue of the non differentiability of the prior at zero. The prior above is such that the MAP estimates will tend to be sparse i.e. if a large number of parameters are redundant, many components of *β *will be zero. Details of the algorithm are given below.

## Results

### Algorithm

The EM algorithm for the general problem defined above can be described with the following steps.

#### Step 1

Set n = 0, initialise *φ*^(0)^, *β*^(0) ^and set tolerance parameters ε, ε_1 _and ε_2 _equal to 10^-4 ^(say). Choose values of k and *δ *in the prior (*k *= 0 and *δ *= 0 often work well in practise).

#### Step 2

For n ≥ 0, perform the E step by computing the conditional expectation *d*^(*n*) ^= (*E*{*ν*^-2^|*β*^(*n*)^, *k*, *δ *})^-0.5^

and

(7) $$\begin{array}{lll} Q(\beta |\beta^{\left(n\right)},\phi^{\left(n\right)}) & = & \text{E}\{\log p(\beta ,\phi ,\nu |y)|y,\beta^{\left(n\right)},\phi^{\left(n\right)}\}\\ & = & L(y|\beta ,\phi^{\left(n\right)})-0.5(||\beta /d^{\left(n\right)}|{|}^{2}) \end{array}$$

where L is the log likelihood function of *y*. Here, and in the following, we adopt the convention that for any component of *β*_*n *_which is zero, the corresponding component of *d*_*n *_is by definition also zero and 0 = 0/0. More details of the derivation of (7) are given in Appendix 1 in the supplementary information.

#### Step 3

Perform the M step, i.e. maximise *Q *in (7) as a function of *β*. This can be done with Newton Raphson iterations as follows. Set *β*_0 _= *β*^(*n*) ^and for r ≥ 0, *β*_*r*+1 _= *β*_*r *_+ *α*_*r *_*η*_*r *_where α_r _is chosen by a line search algorithm to ensure Q(*β*_r+1_|*β*^(n)^, φ^(n)^) > Q(*β*_r_|*β*^(n)^, φ^(n)^) and

(8) $$\eta_{\text{r}}=\Delta (d^{\left(n\right)}){\left[Y_{n}^{T}B_{r}Y_{n}+I\right]}^{-1}(Y_{n}^{T}\frac{\partial L}{\partial \mu_{r}}-\frac{\beta_{r}}{d^{\left(n\right)}})$$

Here, *Y*_*n *_= *X *Δ (*d*_*n*_), $B_{r}=-\partial^{2}L/\partial \mu_{r}^{2}$ and *μ *_*r *_= *X β *_*r*_. Stop when some convergence criterion is satisfied e.g |*β *_*r *_- *β *_*r*+1_| <*ε*, and let β * be the value of *β*_*r*+1 _when iterations are terminated. Note that in many cases (8) is simply a form of iteratively reweighted (and rescaled) ridge regression.

#### Step 4

Update parameter estimates as follows. First eliminate parameters whose values are "too" small. Let $S=\{j:|\beta_{j}^{*}|>\max(|\beta_{k}^{*}|\varepsilon_{1},k\;in\;1,...,p)\}$ and define

$$\beta_{i}^{\left(n+1\right)}=\{\begin{array}{l} \beta_{i}^{*},i\in S\\ 0,otherwise \end{array}.$$

Second, choose *φ*^(*n*+1) ^= *φ*^(*n*) ^+ *κ *_*n *_(φ * - *φ*^(*n*)^) where φ * satisfies $\frac{\partial }{\partial \phi }L(y|\beta^{\left(n+1\right)},\phi )$ and *k*_*n *_is a damping factor such that 0 <*κ *_*n *_≤ 1.

#### Step 5

Check convergence. If |*β *^(*n*+1) ^- *β *^(*n*)^| <*ε *_2 _then stop, else set n = n+1 and go to step 2 above. *End of algorithm*.

For the general case modifications are required if the regularised matrix in (8) is indefinite.

Note that $\frac{\partial^{2}L}{\partial^{2}\mu_{r}}$ in step 4 above can also be replaced by its expectation $\text{E}\{\frac{\partial^{2}L}{\partial^{2}\mu_{r}}\}$ which will be at least negative semi definite. Negative definite (block) diagonal approximations to the second derivative will also generate ascent directions if used in the M step.

### Implementation

The prior distribution discussed here places much more weight on parameters being zero than is customary. There are many issues involved in the practical implementation of the procedure outlined above. Some of these issues are discussed below.

### Initialisation

In general the posterior can have many local maxima so a critical part of the algorithm is the intialisation. Another issue is that initial values too close to zero may also result in iterations converging to *β *= 0.

A good initial value is one for which the likelihood function attains, or is very close to its global maximum. Intuitively, this means we start at a point where the fit to the observed data is the best possible. To make progress from such an initial value, the algorithm can only decrease the second term in Equation (7) by making one or more components of *β *smaller. (Note that the second term of (7) could be interpreted as a collection of pseudo t-statistics.) From such an initial value we can think of the algorithm as maintaining the best fit to the data possible whilst diminishing the importance of and eventually removing parameters from the model. Parameters which can be totally removed from the model without affecting the optimal fit are likely to disappear first until a trade off between model complexity and goodness of fit, as measured by the likelihood function, begins as iterations continue.

For models in the exponential family class, for example generalised linear models, such an initial value can easily be obtained by performing a ridge regression of a transformed and possibly slightly perturbed version of the response vector *y*, see the supplementary information for more details.

### The E step

The components of the conditional expectation required in (7) are given by the following expression

(9) $$E\{\nu_{i}^{-2}|\beta_{i}^{\left(n\right)},k,\delta \}=\frac{\delta }{\left|\beta_{i}^{\left(n\right)}\right|}\frac{K_{3/2-k}(\delta |\beta_{i}^{\left(n\right)})|)}{K_{1/2-k}(\delta |\beta_{i}^{\left(n\right)})|)}$$

for i = 1,..., p, where *K *denotes the modified Bessel function of the third kind and $\delta =\sqrt{2/b}$. The function *K *is a standard function in the R package [11], see also Zhang and Jin [12] for stand alone code. A sketch of the derivation of the above result is given in Appendix 2 in the supplementary information.

Some useful special cases of (9) are:

k = 1

(10) $$E\{\nu_{i}^{-2}|\beta_{i}^{\left(n\right)},k=1,\delta \}=\delta /|\beta_{i}^{\left(n\right)}|$$

k = 0

(11) $$E\{\nu_{i}^{-2}|\beta_{i}^{\left(n\right)},k=0,\delta \}=1/|\beta_{i}^{\left(n\right)}{|}^{2}+\delta /|\beta_{i}^{\left(n\right)}|$$

### The M step

Let *p*^(*n*) ^denote the number of parameters which are currently nonzero at iteration number *n*. We can use the same matrix identity referred to above to obtain expressions for (8) which require inversion of matrices of size min (N, *p*^(*n*)) ^or less.

For *p*^(*n*) ^≤ N use

(12) $$\eta_{\text{r}}=\Delta (d^{\left(\text{n}\right)}){\left[{\text{Y}}_{\text{n}}^{\text{T}}{\text{B}}_{\text{r}}{\text{Y}}_{\text{n}}+\text{I}\right]}^{-1}({\text{Y}}_{\text{n}}^{\text{T}}\frac{\partial L}{\partial \mu_{r}}-\frac{\beta_{\text{r}}}{d^{\left(\text{n}\right)}})$$

and for *p*^(*n*) ^> N use

(13) $$\eta_{\text{r}}=\Delta (d^{\left(\text{n}\right)})[\text{I}-{\text{Y}}_{\text{n}}^{\text{T}}{{\text{(Y}}_{\text{n}}{\text{Y}}_{\text{n}}^{\text{T}}+{\text{B}}_{\text{r}}^{-\text{1}})}^{-1}{\text{Y}}_{\text{n}}]({\text{Y}}_{\text{n}}^{\text{T}}\frac{\partial L}{\partial \mu_{r}}-\frac{\beta_{\text{r}}}{d^{\left(\text{n}\right)}})$$

Note that (12) appears to require the inversion of a *p *by *p *matrix, however the calculation can be done by inverting a *p*^(*n*) ^by *p*^(*n*) ^matrix since *p*-*p*^(*n*) ^columns of *Y*_*n *_are identically zero, see the definition of *Y*_*n *_in Equation (8). By partitioning *Y*_*n*_, *β*_*r*_and *d*^(*n*) ^into conformable zero and non-zero components (12) and (13) can be calculated efficiently. In fact it is only necessary to calculate *η*_*r *_for parameters which are currently non-zero.

When the number of parameters *p*^(*n*) ^in the model becomes less than N the size of the matrices being inverted becomes *p*^(*n*) ^by *p*^(*n*) ^and continues to decrease as more parameters are eliminated from the model. Note that the algorithm can be implemented to be O(min(N^3^, p^3^)).

### Convergence

In practice the algorithm converges rapidly. To see the reason for this, differentiate (7) with respect to *β *to obtain

(14) $$\frac{\partial Q}{\partial \beta }=\frac{\partial L}{\partial \beta }-\frac{\beta }{{\left(d^{\left(n\right)}\right)}^{2}}$$

By the definition of the algorithm in Section 2, *β *^(*n*+1) ^is defined so that the left hand side of (14) is zero. Hence if the sequence (*β*^(*n*)^, *φ*^(*n*)^) converges

$${\left(d^{\left(n\right)}\right)}^{2}(\frac{\partial L}{\partial \beta^{\left(n+1\right)}})=\beta^{\left(n+1\right)}.$$

For the NG prior, using Abramowitz and Stegun [13], section 9.6.9, it can be shown that for small beta and 0 ≤ *k *≤ 0.5 we have

(15) *E*{*ν*^-2^|*β *^(*n*)^}~*b*(*k*)/|*β *|^2 ^

and for 0.5 ≤ *k *≤ 1 we have

(16) *E*{*ν*^-2^|*β *^(*n*)^}~*c*(*k, δ*)/|*β *|^(3-2*k*) ^

where *b*(*k*) and *c*(*k*, *δ*) are constants. It follows that the rate of convergence to zero of the outer iteration in the EM algorithm is quadratic for 0 ≤ *k *≤ 0.5 and varies from quadratic to linear as *k *varies from 0.5 to 1.

### Multiple solutions

Multiple maxima of the posterior can be explored by sequentially running the algorithm and deleting selected variables from consideration in the next run. This often produces classes of models with similar predictive performance which can be used to provide alternative or expanded interpretations. Predictions using these models can also be combined by majority voting schemes or model averaging.

When N <*p *the likelihood has flat spots as can be seen from the relation

(17) *X β *= *X *(*β *+ *r*)

where r is orthogonal to the row space of *X*. Using (17), given a starting value *β*_0 _with likelihood value close to the global maximum, random points with this same property can be generated as follows. Generate a p by 1 vector of random variables *n*, compute

(18) $$\begin{array}{l} r=(I-X^{T}{\left(XX^{T}\right)}^{-1}X)n\\ \beta_{r}=\beta_{0}+r \end{array}$$

It is easy to see that *β*_*r *_has the same likelihood value as *β*_0_.

### Forcing variables into the model

The algorithm can be easily modified to force variables into the model (eg intercepts) by simply not penalising certain coefficients.

### Selection of hyperparameters

In the prior discussed here, the choice *k *= 0, *δ *= 0 (i.e no tuning parameters) works surprisingly well in many cases, giving very sparse models with small cross validation errors. However, this prior can sometimes lead to the elimination of all variables. In such cases cross validated errors computed over a grid of *k *and *δ *values can provide guidance in selecting the hyperparameters. Often, setting *δ *= 0 and just computing cross validated errors over a grid of values of *k *works well. Note that any process for assessing the quality of the predictions from a model chosen in this way should explicitly include this selection process to avoid selection bias. We will expand on this below.

### Implementing multiclass logistic regression

To implement the algorithm for a particular model simply requires expressions for the first two derivatives of the likelihood function. See the supplementary information for details for multiclass logistic regression.

### Enlarged sets of predictors

As mentioned earlier, enlarged sets of predictor variables for biological interpretation can be identified by running the algorithm multiple times and removing variables previously selected from consideration. An alternative strategy, which can identify sets of important highly correlated variables is to define a new *X *matrix by clustering the columns of the original matrix *X *and taking means of the clusters [14].

### Other models

It can be shown that the algorithm still applies if the likelihood function in (6) is replaced by some other goodness of fit criterion. For example, linear kernel support vector machines can be implemented with the above algorithm (and a Gaussian prior) by using the penalized hinge loss formulation and noting that

|1 - *x*|_+ _= lim *γ *^-1 ^log (1 + exp(*γ *(1 - *x*))

where |1 - *x*|_+ _= max (-(1 - *x*),0) and the limit means *γ *→ ∞, see for example [15]. Using the above criterion with the normal gamma prior, we can also fit the L1 penalised support vector machine (k = 1) and a more strongly penalised version with no tuning parameters (k = 0, *δ *= 0).

A minor modification to the E step enables L1 linear regression with L1 constraints (k = 1) on the parameters to be fitted by the algorithm. There is also a penalised version of L1 regression with no tuning parameters (k = 0, *δ *= 0).

Note that we do not need to use linear or quadratic programming to fit these models. More details will be reported elsewhere.

### Testing

We give some examples of fitting models to data with orders of magnitude more variables than samples using various likelihood functions below. To eliminate selection bias [16] in our assessment of predictions, we validate results either on a totally independent data set, or through the use of n fold cross validation in such a manner that for each fold the hold out sample plays no role whatsoever in the formulation of our prediction [17]. For models with no hyperparameters this means that for each fold the simultaneous model fitting and variable selection is redone and predictions then made for the holdout data. For models with hyperparameters, any cross validation necessary to estimate these parameters is also redone for each fold. Where necessary, we will refer to the above as "including the variable selection or hyperparameter selection process in the cross validation". In the examples below, with the possible exception of the ordered categorical data example, selection bias has been accounted for by the above methods. The results for all the examples are for very sparse models found by the algorithm.

### Smoking Data

For our first example we use the gene expression data (Affymetrix U133A chips) from Spira et al. [18]. We analyse a subset of the data consisting of 34 current smokers and 23 who have never smoked. We treat smoking status as a binary "response" and search for genes which are predictive of this "response" in a logistic regression model. The number of variables (genes) in this problem is 22283.

For the default values *δ *= 0 and k = 0, the algorithm discovers a model with three genes for the full dataset. The 10 fold cross validated error rate, calculated as mentioned above, is 0.07. The corresponding misclassification table is given in the supplementary information.

For illustrative purposes, we also computed the 10 fold cross validated error rates for a grid of values of the hyperparameters *b *and *k *in the NG sparsity prior, see supplementary information. The smallest value was 0.018, corresponding to one misclassification. For *k *= 0.6, and *δ *≈ 0 (*δ *= 0 is a limiting case as b → ∞) the apparent error rate was 0.035. For this combination, neighbouring grid values had similar error rates so the specific values of k and b are not critical. The model involved 5 genes. Including the choice of k in the cross validation (*δ *= 0) gave a cross validated error rate of 0.052. Including the choice of both hyperparameters in the cross validation did not increase this error rate.

For comparison purposes we also used a support vector machine [19-21] with recursive feature elimination [22] to classify this data. The best model contained 8 genes and had an apparent (i.e variable selection step not included in the validation) zero cross validated error rate. Three of the genes were common to those found by our algorithm. When the variable selection process was included in the cross validation this error rate increased to about 10%.

With random forests [23], using the top 5 variables ranked by standardised variable importance gave an out of bag error rate of 0.052. Including the variable selection step in the cross validation had neglible effect on this error rate.

Enlarged lists of discriminating genes can be found by our algorithm by deleting the genes found and rerunning the algorithm. This can be repeated multiple times until there is no longer any discriminating power in the resulting models. For this data set almost all of the genes found by SVM and random forests appear in this expanded list.

When classes appear to be linearly separable, experience with a variety of data sets with small to moderate sample sizes suggests that the methods described in this paper give comparable and sometimes marginally better results to those obtained by support vector machines or random forests. The main advantage is that there is no need to do additional steps such as recursive feature elimination or pruning with variable importance to arrive at a sparse model. The other advantage is that many different types of models can be handled in this one framework.

### Ordered Categorical prostate cancer data

We analysed some data reported by Tomlins et al. [24] on stages of prostate cancer. The data set consisted of 20,000 gene expression measurements obtained from 104 "samples" classified into a number of ordered categories. For illustrative purposes here we analyse the 86 observations with categories 1-benign, 2-cancer, 3-metastasized. We omitted 97 genes which had all values missing. The remaining missing values were imputed using a simple model involving the observed row and column means of the data matrix. To access the data in its original form see the supplementary information. Using the algorithm in section 2 we fit the odds continuation ratio model [25] with

$$\begin{array}{cc} \text{logit}(\frac{p_{ig}}{p_{ig}^{*}})=\theta_{g}+x_{i}^{T}\beta , & 1<g\leq G \end{array}$$

where *p*_*ig *_denotes the probability that observation i belongs to class g, $p_{ig}^{*}=\sum_{h=1}^{g}p_{ig}$ and $x_{i}^{T}$ denotes the i^th ^row of the expression data matrix *X*. Here G = 3.

Applying the algorithm to this data set produces a 4 gene model with the cross validated confusion matrix in Table 1 below.

**Table 1:** Prostate cancer example (10 fold) cross validated confusion matrix

	Predicted			
Observed	Benign	Cancer	Metastasized	Proportion correct

Benign	18	3	0	0.85
Cancer	5	38	2	0.84
Metastasized	0	8	12	0.60

Note the lower accuracy for class 3. To see if this could be improved we did a weighted analysis with observation weights inversely proportional to the number of observations in each class, but with the resulting weights for class 3 being multiplied by 2. Rerunning the algorithm with these weights gave the results in Table 2 below.

**Table 2:** Prostate cancer example- weighted analysis (10 fold) cross validated confusion matrix

	Predicted			
Observed	Benign	Cancer	Metastasized	Proportion correct

Benign	16	5	0	0.76
Cancer	4	36	5	0.80
Metastasized	0	4	16	0.80

This time a model with 5 genes was identified of which three were in common with the previous model.

We should be cautious about this last model and ideally it should be validated with independent data, however it serves to illustrate the application of our methodology to ordered categorical data. Other methods such as, support vector machines and random forests do not take into account any ordering present in categorical variables.

### Multiclass Leukaemia data

To illustrate an application of the multiclass logistic regression model we consider the Leukaemia dataset reported by Ross et al. [26]. The data consists of microarray measurements from Affymetrix U133 A and B chips. There were104 individuals classified into 6 sub types of leukaemia (the "other" class is omitted). We do a probe level analysis so p = 497467 i.e. there are roughly half a million variables. The class names are (1 – T-ALL, 2 – E2A-PBX1, 3 – MLL-rearrangement, 4 – BCR-ABL, 5 – TEL-AML1, 6 – Hyper diploid).

Applying the methodology described above, the leave one out cross validation error is 0.048. The cross validated misclassification matrix is given in the supplementary information.

The method identified 5 probes which appear to be useful for sub-typing leukaemia. Using random forests [23] (with no additional variable selection step) the out of bag error rate is 0.019 using over 3000 probes and 0.096 for a model using the top ten probes ranked by standardised variable importance. This latter figure did not increase by including the variable selection step in the cross validation.

### Survival analysis

To illustrate the use of our method with survival data we fit a Cox proportional hazards model [2] to the Lymphoma data set reported by Dave et al. [27]. See the supplementary information for access details.

This data set consists of 44928 "gene" (probe set) expression measurements from Affymetrix U133A and B chips on 191 patients. Censored survival times were available for 187 of these individuals. In Dave et al. [27] the data was divided into a training set of 95 observations and a validation set of 96 observations. Allowing for missing survival times the training set has 93 individuals and the validation set 94 individuals. Note that the validation set has censored observations.

Applied to the training data, we used the algorithm of section 2 to identify 3 genes as being potentially associated with survival. Using the baseline survival function and the coefficients of the linear predictor estimated from the training data we obtained (predicted) survival curves for each individual in the validation data set. From these predicted survival curves we calculated the probability of each individual in the validation set dying in a defined set of time intervals and computed the expected number of deaths in each of these intervals. We then calculated the observed number of deaths in these intervals for the validation data set. We included the censored individuals in this step by computing the conditional probability of a death (using the predicted survival function) in any interval given the time was greater then the censoring time. As a consequence the "observed" counts have non-integer values. Table 3 below shows the results for the model with three genes. Taking the L1 norm of the observed minus expected counts on the *validation *data as a statistic, we then generated a null distribution for this statistic by permuting the rows of the X matrix for the *training *data 200 times, rerunning the algorithm each time and making a prediction on the validation data. The p value of our observed statistic was about 0.2, which suggests the support for this model is not strong.

**Table 3:** Observed and expected counts in validation set for 3 gene model

**Time interval**	0–5 yrs	5–10 yrs	10–15 yrs	15–20 yrs	> 20 yrs
**Observed**	28.74	21.67	16.93	13.14	13.51
**Expected**	28.02	20.12	15.58	15.15	15.13

In their paper, using their survival signature analysis method, Dave et al. (2004) computed a survival index based on over 60 gene expression measurements. Repeating the calculation above for their survival index on the validation data gave Table 4 below.

**Table 4:** Observed and expected counts in validation set for Dave et al. (2004) survival index using over 60 genes

**Time interval**	0–5 yrs	5–10 yrs	10–15 yrs	15–20 yrs	> 20 yrs
**Observed**	28.14	20.33	18.09	13.42	14.02
**Expected**	25.82	22.55	19.31	15.27	11.06

Note that on the basis of the L1 norm statistic, the 3 gene fit has a smaller L1 norm.

### Ethnicity and sex – Perlegen SNP data

We now illustrate how the method scales up to datasets with millions of variables. In a recent article, Hinds et al. [28] report on the collection and analysis of a large data set in which 71 individuals were genotyped for over 1.5 million single-nucleotide polymorphisms (SNPs). Ethnicity and sex information for each of the 71 individuals was also recorded. Using only SNPs on chromosomes 1–22, the methods in this paper identified two SNPs which classified sex and three SNPs which classified ethnicity. The identified SNPs were validated with the Hapmap data set [29]. A publication giving more details about these results is in preparation.

## Discussion

Although in the above we have not provided details of the genes (variables) in the models presented in the above examples, in cases when the gene function is known, the selected genes have a biologically meaningful function in the context of the dataset being analysed. Specifically, for the Smoking data we saw genes appearing in networks associated with biological themes that we'd expect from an assault such as smoking on tracheal epithelial cells. Many of these are well documented in the literature, e.g. xenobiotic metabolism (P450 family of genes, CYP1A1), genes associated with immune function (complement system, C3) and inflammatory response. In addition there were genes involved in the early-immediate stress response (fos, jun, glutathione) which is expected from a toxic challenge to cells. Likewise, the genes in the leukemia classifier showed links with genes related to various aspects of the cell cycle, DNA repair, DNA replication and check-point controls as well as genes involved in cell growth and proliferative responses. Finally for the Perlegen SNP data the ethnicity classifier used a SNP which was associated with a gene which codes for skin colour.

Biological interpretations like the above have also been reflected in our experience with these methods over a number of years.

Concerning the computer time required to analyse these examples, on a 2.2 GHz machine, the two class problem with 20,000+ variables ran in under one minute. The six class problem with roughly 500,000 variables took about half an hour to run, and the two class problem with 1.5 million SNPs (three million variables) ran in about an hour and a half. Examples were run in R with no particular optimisation of the code. The times for the SNP example could most likely be reduced by the use of sparse matrices.

## Conclusion

Using a sparsity prior or sparsity penalty in conjunction with a likelihood function is a powerful approach to finding parsimonious models for datasets with many more variables than observations. The method is capable of handling problems with millions of variables and makes it possible to fit almost any statistical model with a linear predictor in it to data with more variables than observations.

In the linear case, and where comparison is possible, the methods described in this paper compare favourably with well known methods such as support vector machines and random forests. However, they have the advantage in that variable selection and parameter estimation occur simultaneously and no additional steps are required to obtain a sparse model.

An R library implementing the algorithm described in this paper is freely available for non-commercial use [30].

## Supplementary Material

Additional file 1 Supplementary information. Click here for file
