# Supplementary material for: A general approach to simultaneous model fitting and variable elimination in response models for biological data with many more variables than observations
Source: BMC Bioinformatics. 2008 Apr 15;9:195. doi: 10.1186/1471-2105-9-195 (PMC2390543; doi:10.1186/1471-2105-9-195)
Supplement: Additional file 1 — Supplementary information. [file 1471-2105-9-195-S1.doc]

A general approach to simultaneous model fitting and variable elimination in response models for biological data with many more variables than observations

Supplementary material

by

Harri Kiiveri

CSIRO Mathematical and Information Sciences

### The Leeuwin Centre, Floreat, Western Australia

# **Abstract**

## Background

With the advent of high throughput biotechnology data acquisition platforms such as micro arrays, SNP chips and mass spectrometers, data sets with many more variables than observations are now routinely being collected. Finding relationships between response variables of interest and variables in such data sets is an important problem

akin to finding needles in a haystack.

### **Results**

The major contribution of this paper is to present a unified methodology which allows many common (statistical) response models to be fitted to such data sets. The class of models includes virtually any model with a linear predictor in it, for example (but not limited to), multiclass logistic regression (classification), generalised linear models (regression) and survival models. A rapid algorithm for finding sparse well fitting models is presented. The ideas are illustrated on real data sets with numbers of variables ranging from thousands to millions.

## Conclusion

The method described in this paper enables existing work on response models when there are less variables than observations to be leveraged to the situation when there are many more variables than observations. The method is a powerful approach to finding parsimonious models for datasets with many more variables than observations. The method is capable of handling problems with millions of variables and makes it possible to fit almost any statistical model with a linear predictor in it to data with more variables than observations. The method compares favourably to existing methods such as support vector machines and random Forrests, but has the advantage of not requiring separate variable selection steps. It is also capable of handling data types which these methods were not designed to handle

Note: In the following, section titles are the same as those of the paper which refer to supplementary information.

BACKGROUND

**Some insights from a simple linear regression example**

Following Fan and Li (2001), to develop some insight into the algorithm we consider a linear regression model

$$y=X\beta+\varepsilon$$

where X is N by p, full rank, with p$\leq$ N and$\varepsilon\sim N(0,\sigma^{2}I)$. A Taylor expansion about the point $\hat{\beta}=(X^{T}X)^{-1}X^{T}y$ shows that

$$\begin{matrix} P=-\{\log L+\log p(\beta)\} \\ =c(\sigma^{2})+(\beta-\hat{\beta})^{T}(X^{T}X\sigma^{-2})(\beta-\hat{\beta})-\log p(\beta) \\ =c(\sigma^{2})+(\beta-\hat{\beta})^{T}V(\hat{\beta})^{-1}(\beta-\hat{\beta})-\log p(\beta) \\ \end{matrix}$$

where $V(\hat{\beta})$ denotes the variance of$\hat{\beta}$. For $X$orthogonal (22) becomes

$$P= c(\sigma^{2})+\frac{1}{2}\sum_{i=1}^{p} \{\frac{(\beta_{i}-\hat{\beta}_{i})^{2}}{s_{i}^{2}}-\log p(\beta_{i})\}$$

where $s_{i}^{2}$is the i^th^ diagonal element of$\sigma^{2}(X^{T}X)^{-1}$. From the form of (3) we see that P can be minimised by independently minimising each term in the sum. When $p(\beta)$ is derived from a hierarchical prior as in (1), we can write

$$\frac{\partial P}{\partial\beta_{i}}=\frac{(\beta_{i}-\hat{\beta}_{i})}{s_{i}^{2}}+\beta_{i} E\{\nu_{i}^{-2}|\beta_{i},k,\delta\}$$

where $E$denotes conditional expectation. The derivation of the second term in (4) is given in Appendix 3. Note that this term could be regarded as a pseudo T-statistic.

Figure 1 gives a plot of a single component of (3) with the normal gamma prior, $s_{i}^{2}=1$

and the usual regression estimate is $\hat{\beta}_{i}$=4.

Figure 1: Plot of penalised likelihood function for single parameter and$\hat{\beta}_{i}$=4

From (4) we get the MAP (penalised likelihood) estimate satisfying the equation

$\beta_{i}=\frac{\hat{\beta}_{i}}{(1+s_{i}^{2}E\{\nu_{i}^{-2}|\beta_{i},k,\delta\})}$ .

Since the numerator is greater than one this implies that the penalised likelihood estimate is shrunken towards zero when compared to the usual regression estimate.

Threshholding

Equation (4) can be written as

$$s_{i}^{2}\frac{\partial P}{\partial\beta_{i}}=h(\beta_{i})-\hat{\beta}_{i}$$

where$h(\beta_{i})=\beta_{i}(1+ s_{i}^{2}E\{\nu_{i}^{-2}|\beta_{i},k,\delta\})$. Note that h is an odd function. A plot of this function for several values of k and $\delta$ is given in Figure 2 below.

Figure 2: Plot of function h for various values of $\delta$and k

In general, if the function h has a minimum at $\beta_{0}>0$ with the property that $h(\beta_{0})>0$, then from (26) whenever $|\hat{\beta}_{i}|<h(\beta_{0})$ the minimum of P occurs at $\beta=0$ since P is increasing for $0<\hat{\beta}_{i}<h(\beta_{0})$ and decreasing for $-h(\beta_{0})<\hat{\beta}_{i}<0$. Hence there is a threshold at $h(\beta_{0})$

and absolute values of the regression estimate below this value give a penalised likelihood estimate of zero. This is the case for the NG prior. Figure 3 below plots the thresholding function for several values of k and$\delta$.

Figure 3: Plot of thresholding functions for the normal gamma prior

The threshold values for the three cases in the above plot are 2, approximately 3.32 and 2 respectively. Hence for the case k=0 and$\delta=0$, thresholding occurs whenever the regression estimate is less than 2 in absolute value.

Concerning initialisation of the algorithm for this linear regression case, from Figure 1 we can see that starting from the global maximum of the likelihood function (the regression estimate) an optimisation algorithm with a sensible line search will find the relevant minimum of minus the penalised likelihood function when the regression estimate is larger than the threshold value. A similar figure for the case that the regression estimate is less than the thresholding value shows that the minimiser of the penalised likelihood function will converge to zero.

Note that whilst the above discussion gives some insight into what the general algorithm is doing, the case of p>>n is more complex as there are potentially many full rank subsets of the columns of $X$ and the columns of $X$ will not be orthogonal.

CONTEXT AND METHODS

Alternative sparsity priors or penalties

Fan and Li (2001) discuss three desirable properties of thresholding functions, namely unbiasedness, sparsity and continuity, and suggest a penalty function, the smoothly clipped absolute deviation (SCAD) penalty which has these three properties. The normal gamma prior (penalty) has thresholding functions which typically satisfy their first two properties but not the third. However, experience with numerous examples has shown that this set of penalty functions works well for problems with p>>n. When there are strong effects present in the data, parameter estimates tend not to be close to thresholding values where the discontinuity lies.

Using the results in Appendix 3, the SCAD penalty function can be converted to a nominal conditional expectation “equivalent” using

$$E\{\nu^{-2}|\beta\}=\frac{1}{\beta}\frac{\partial\pi}{\partial\beta} = \frac{1}{ \left|\beta\right|} \frac{\partial\pi}{\partial\left|\beta\right|}$$

where $\pi$ denotes minus log of the SCAD penalty function. The algorithm described in the paper can then be used to fit models with the SCAD penalty. For numerical reasons this “expectation” needs to be truncated below some small value.

Fan and Li also describe an algorithm for computing estimates from penalised likelihood functions. When the penalty function is derived from a hierarchical prior, their algorithm is an EM algorithm.

An alternative to the NG prior and the SCAD penalty is discussed in Griffin and Brown (2005).

RESULTS

Algorithm initialisation

Let *h* be the link function in a generalised linear model. If p ≤ N compute initial values $\beta^{(0)}$ using

$\beta^{(0)}=(X^{t}X+\lambda I)^{-1}X^{T}h(y+\zeta)$ (8)

where the ridge parameter λ satisfies 0 < λ ≤ 1 (say) and ζ is small and chosen so that the link function *h* is well defined at y+ζ. If p > N, to keep matrix sizes manageable, Equation (8) can be rewritten using a familiar matrix identity in Rao (1973). In this case compute initial values *β*^(0)^ by

$\beta^{(0)}=\frac{1}{\lambda}(I\text{ }-X^{T}(XX^{T}+\lambda I)^{-1}X)X^{T}h(y+\zeta)$ . (9)

For example, for logistic regression, $\zeta$could have the value -0.001 when *y*=1 and +0.001 when y=0. In this case the function g is the logit function. The rationale for the perturbation is that the global maximum of the likelihood function would have probabilities estimated as one when y is one and zero when y=0. Unfortunately the logit function is undefined at these values so we perturb them slightly and then do a ridge regression to find a value of beta which gives probabilities close to these values.

Writing $\hat{y}$ for the ridge regression predictor of y and using the singular value decomposition $X^{T}=U\Omega V^{T}$we can show that

$$||y-\hat{y}||^{2} = \sum_{i=1}^{n} (\frac{\lambda}{\lambda+\omega_{ii}^{2}} )^{2}z_{i}^{2}$$

where $\omega_{ii}$ are the singular values of X and $z=V^{T}y$. If X is full rank, lambda can be chosen in the above expression to make the norm small. For example, given $\varepsilon$ “small”, writing $\omega^{*}=\min\{\omega_{ii}^{2}:i=1,...,n\}\neq0$ , $z^{*}= \max\{z_{i}^{2}:i=1,...,n\}$ and $a=\sqrt{\varepsilon/(nz^{*})}$then $\lambda\leq a\delta^{*}/(1-a)$ implies that $||y-\hat{y}||^{2} \leq\varepsilon$.If the singular values are large then a value of 1 for lambda will often be sufficient.

Note that the initial value calculations can be performed by inverting matrices of the size min(n,p). The matrix identity used in deriving (9) is also useful during the M step to avoid the calculation and inversion of large matrices. Considering that we will apply this algorithm to data sets with millions of variables, this is an important point.

## Implementing multiclass logistic regression

To implement the algorithm for a particular model simply requires expressions for the first two derivatives of the likelihood function. In this section we present some details which enable the implementation of the algorithm for the multiclass classification problem.

Consider a set of N individuals classified into G$\geq$2 classes with associated explanatory variables measured in the N by *p* matrix X with i^th^ row$x_{i}^{T}$. Here the response y is a vector of class labels for each individual. Define

and

where $p_{ig}$ denotes the probability that individual i is in class g.

For the multiclass logistic regression model we write

$p_{ig}=\frac{\exp(x_{i}^{T}\beta_{g})}{\left(\sum_{h=1}^{G} \exp(x_{i}^{T}\beta_{h})\right)}$ (10)

From (10) the log likelihood function is

$\begin{matrix} \log L= \sum_{i=1}^{N} \left(\sum_{g=1}^{G} e_{ig}\log(p_{ig})\right) \\ = \sum_{g=1}^{G} e_{g}^{T}\log(p_{g}) \end{matrix}$ (11)

Writing $\mu_{g}=X\beta_{g}$, from (11) it is easy to show that

(12)

Setting$\mu^{T}=(\mu_{1}^{T},...,\mu_{G}^{T})$, the above expressions can be used to implement the algorithm in Section 2. In practice we use a block diagonal approximation to the second derivative matrix as this allows the ascent directions in the M step to be calculated sequentially over the classes without storing matrices larger than N by *p*. Our experience has been that this approximation has worked very well over a range of problem sizes.

To implement the general algorithm for other likelihood functions simply requires the calculation of the two derivatives of the likelihood function in a similar manner as above.

**Smoking data example**

|  | Predicted class | |
| --- | --- | --- |
| True class | Smoker | non smoker |
| smoker | 33 | 1 |
| non smoker | 3 | 20 |
|  |  |  |

Table s.1: Cross validated misclassification table (k=0, δ=0)

|  | b - scale parameter in prior ($\delta=\sqrt{(2/b)}$) | | | | | | | | | |
| --- | --- | --- | --- | --- | --- | --- | --- | --- | --- | --- |
| k | 0.01 | 0.1 | 1 | 10 | 100 | 1000 | 1e+04 | 1e+05 | 1e+06 | 1e+07 |
| 0 | 0.088 | 0.053 | 0.070 | 0.070 | 0.070 | 0.053 | 0.053 | 0.088 | 0.070 | 0.070 |
| 0.1 | 0.053 | 0.070 | 0.053 | 0.088 | 0.070 | 0.088 | 0.088 | 0.070 | 0.088 | 0.088 |
| 0.2 | 0.070 | 0.088 | 0.070 | 0.070 | 0.088 | 0.088 | 0.088 | 0.123 | 0.053 | 0.105 |
| 0.3 | 0.053 | 0.070 | 0.088 | 0.070 | 0.053 | 0.035 | 0.088 | 0.070 | 0.070 | 0.070 |
| 0.4 | 0.035 | 0.070 | 0.053 | 0.070 | 0.088 | 0.053 | 0.053 | 0.070 | 0.070 | 0.053 |
| 0.5 | 0.035 | 0.053 | 0.070 | 0.035 | 0.070 | 0.053 | 0.070 | 0.035 | 0.053 | 0.053 |
| 0.6 | 0.088 | 0.035 | 0.053 | 0.053 | 0.070 | 0.053 | 0.070 | 0.035 | 0.035 | 0.035 |
| 0.7 | 0.035 | 0.053 | 0.035 | 0.035 | 0.035 | 0.053 | 0.053 | 0.035 | 0.035 | 0.035 |
| 0.8 | 0.035 | 0.035 | 0.053 | 0.053 | 0.053 | 0.088 | 0.053 | 0.018 | 0.053 | 0.035 |
| 0.9 | 0.035 | 0.035 | 0.035 | 0.053 | 0.035 | 0.035 | 0.018 | 0.035 | 0.018 | 0.035 |
| 1 | 0.035 | 0.035 | 0.035 | 0.035 | 0.035 | 0.035 | 0.018 | 0.018 | 0.018 | 0.035 |

Tables s.2: Cross validated error rates for a grid of b and k values

Leukaemia example

|  | PREDICTED CLASS | | | | | |
| --- | --- | --- | --- | --- | --- | --- |
| TRUE CLASS | T-ALL | E2A-PBX1 | MLL | BCR-ABL | TEL-AML | HYPERDIP |
| T-ALL | 12 | 0 | 2 | 0 | 0 | 1 |
| E2A-PBX1 | 0 | 18 | 0 | 0 | 0 | 0 |
| MLL | 0 | 0 | 17 | 0 | 0 | 0 |
| BCR-ABL | 1 | 0 | 0 | 19 | 0 | 0 |
| TEL-AML | 0 | 0 | 0 | 0 | 20 | 0 |
| HYPERDIP | 1 | 0 | 0 | 0 | 0 | 13 |

**Table s.3:** **Cross validated misclassification matrix for leukaemia data (k=0, δ=0)**

**Data**

Links to original data and associated R workspaces:

1. Smoking data

The raw data can be found at http://pulm.bumc.bu.edu/aged/download.html

1. Prostate cancer data

The raw data can be found at

[**http://www.ncbi.nlm.nih.gov/geo/query/acc.cgi?acc=GSE6099**](http://www.ncbi.nlm.nih.gov/geo/query/acc.cgi?acc=GSE6099)

by clicking on the Series Matrix File(s) link.

1. Leukaemia data

Primary data is available at <http://www.stjuderesearch.org/data/ALL3>

1. SNP data

The raw data can be found at http://genome.perlegen.com/browser/download.html

1. Follicular lymphoma survival data

The raw data is available for download from http://llmpp.nih.gov/FL/

**Appendices**

Appendix 1: Derivation of equation 7

Using the same notation and equation numbering as in the paper, from equation (6), namely

$$p\left(\beta, \phi,\nu\left|y\right.\right) \alpha L\left(y\left|\beta, \phi\right.\right)p\left(\beta\left|\nu\right.\right)p\left(\nu\right)$$

we have

$$\begin{matrix} Q(\beta|\beta^{(n)},\phi^{(n)})\text{ }= E\{\text{ }\log\text{ }p(\beta,\phi,\nu|y)\text{ }|\text{ }y,\beta^{(n)},\varphi^{(n)}\} \\ \text{ }=\text{ } E\{\log L\left(y\left|\beta, \phi\right.\right)+ \log p\left(\beta\left|\nu\right.\right)+\log p\left(\nu\right)|\text{ }y,\beta^{(n)},\varphi^{(n)}\} \end{matrix}$$

Noting that $p\left(\beta\left|\nu\right.\right)$ is a normal distribution and ignoring terms which do not involve $\beta$ or $\phi$ this last expression can be written as

$$\log L-0.5*\beta^{2}*E\{\nu^{-2}|\text{ }y,\beta^{(n)},\varphi^{(n)}\}$$

and Equation (7) follows on noting that

$d^{(n)} =(E\{\nu^{-2} | \beta^{(n)},k,\delta\})^{-0.5}$.

Appendix 2: Derivation of the conditional expectation in the E step

Suppose $\nu_{i}^{2}$ has a gamma distribution with shape parameter k and scale parameter b i.e.

$p(\nu_{i}^{2})\propto b^{-1}(\nu_{i}^{2}/b)^{k-1}\exp(-\nu_{i}^{2}/b)$.

From the definition of the conditional expectation, writing$\lambda_{i}=\beta_{i}^{2}/2b$, we get

$E\{\nu_{i}^{-2} | \beta_{i}\}= \frac{\int_{0}^{\infty} \nu_{i}^{-2}\nu_{i}^{-1}\exp(-b\lambda_{i}\nu_{i}^{-2})b^{-1}(\nu_{i}^{2}/b)^{k-1}\exp(\nu_{i}^{2}/b)d\nu_{i}^{2}}{\int_{0}^{\infty} \nu_{i}^{-1}\exp(-b\lambda_{i}\nu_{i}^{-2})b^{-1}(\nu_{i}^{2}/b)^{k-1}\exp(\nu_{i}^{2}/b)d\nu_{i}^{2}}$ (A.1)

Rearranging, simplifying and making the substitution $\text{u=ν}_{\text{i}}^{2}\text{/b}$ gives

$\begin{matrix} E\{\nu_{i}^{-2} | \beta_{i}\}= \frac{\int_{0}^{\infty} u^{k-3/2-1}\exp(-(\lambda_{i}/u +u)) du}{b\int_{0}^{\infty} u^{k-1/2-1}\exp(-(\lambda_{i}/u +u) )du} \\ \end{matrix}$ (A.2)

Then using the result

$\int_{0}^{\infty} x^{-b-1}\exp\left[-\left(x+\frac{a^{2}}{x}\right)\right]dx=\frac{2}{a^{b}}K_{b}\left(2a\right)$ (A.3)

where K denotes a modified Bessel function, see Watson (1966), we obtain

$\begin{matrix} E\{\nu_{i}^{-2} | \beta_{i}\} =\sqrt{\frac{2}{b}}\frac{1}{|\text{ }\beta_{i}|}\frac{K_{3/2-k}(2\sqrt{\lambda_{i}})}{K_{1/2-k}(2\sqrt{\lambda_{i}})} \\ =\frac{1}{|\text{ }\beta_{i}|^{2}}\frac{(2\sqrt{\lambda_{i}})K_{3/2-k}(2\sqrt{\lambda_{i}})}{\text{ }K_{1/2-k}(2\sqrt{\lambda_{i}})} \end{matrix}$ (A.4)

and the result follows.

Appendix 3: Identities involving the derivative of the penalty function or prior

Say we have a hierarchical prior

$p(\beta) = \int_{\upsilon^{2}} p(\beta|\nu^{2})p(\nu^{2})d\nu^{2}$ (A.5)

then to be consistent with Fan and Li (2001) we have a penalty function

$\pi(\beta)=-\log(p(\beta))$ (A.6)

It is easy to show the following identity

$\frac{\partial\pi}{\partial\beta} = -E\{\frac{\partial\log(p(\beta|\nu^{2}))}{\partial\beta} | \beta\}$ (A.7)

which for Gaussian $p(\beta|\upsilon^{2})$ gives

$\frac{\partial\pi}{\partial\beta} =\beta E\{\nu^{-2}|\beta\}$ (A.8)

and a bit of rearranging gives us

$E\{\nu^{-2}|\beta\}=\frac{1}{\beta}\frac{\partial\pi}{\partial\beta} = \frac{1}{ \left|\beta\right|} \frac{\partial\pi}{\partial\left|\beta\right|}$ (A.9)

Equation A.9 connects up the use of penalty functions and Hierarchical priors. Clearly if we know the conditional expectation in (A.9) we can compute the quantities required in Fan and Li’s algorithm and vice versa. In fact when the penalty function is derived from a hierarchical prior, Fan and Li’s algorithm is an EM algorithm.

Conversely, for any reasonable penalty function, we can define the “conditional expectation equivalent” using (A.9) and fit the model using the algorithm described in this paper.

**References in Appendices**

Fan, J. and Li, R.Z. (2001). Variable selection via penalized likelihood. Journal of American Statistical Association, 96, 1348-1360.

Griffin, J. E., and Brown, P. J.(2005). Alternative prior distributions for variable selection with very many more variables than observations. Available for download from www2.warwick.ac.uk/fac/sci/statistics/staff/academic/griffin/personal

Watson, G. N.(1966) *A Treatise on the Theory of Bessel Functions,* Second Edition, Cambridge University Press.

**Contacts**

Corresponding author

[harri.kiiveri@csiro.au](mailto:harri.kiiveri@csiro.au)

CSIRO bioinformatics home page

http://www.bioinformatics.csiro.au/
